# Exploring Ultrasonographic Atypical Aspects in Drug‐Resistant Multifocal Chronic Inflammatory Demyelinating Polyneuropathy

**DOI:** 10.1002/brb3.70690

**Published:** 2025-07-20

**Authors:** Angela Puma, Aurora Parrotta, Nicolas Azulay, Andra Ezaru, Michele Cavalli, Mihai Ioncea, Luisa Villa, Nicolae Grecu, Giulia Tammam, Sabrina Sacconi, Simona Maccora, Charles Raffaelli

**Affiliations:** ^1^ Peripheral Nervous System & Muscle Department université cote d'azur Nice France; ^2^ Université Côte d’Azur, Peripheral Nervous System & Muscle Department, Pasteur 2 Hospital, Centre Hospitalier Universitaire de Nice, Nice Nice France; ^3^ Ultrasound Department Côte D'azur University, Nice University Hospital Nice France; ^4^ Neurology Department University Emergency Hospital Bucharest Bucharest Romania; ^5^ Department of Biomedicine, Neurosciences and Advanced Diagnostics (Bi.N.D.) University of Palermo Palermo Italy

**Keywords:** echogenicity, fascicle, multifocal‐CIDP, nerve‐CSA, ultrasound

## Abstract

**Introduction/aims:**

Chronic inflammatory demyelinating polyneuropathy (CIDP) and its variants are characterized by nerve enlargement (NE), particularly in the proximal segments of the median nerve (MN) and cervical roots, as assessed by ultrasound (US). NE is typically moderate and seldom exceeds double the normal size of the cross‐sectional area (CSA). Furthermore, limited knowledge exists regarding the changes in the internal structure of nerves evaluated with high‐frequency ultrasound. This study describes three cases of significant CIDP‐NE assessed with an ultra‐high‐frequency probe (UHF‐US; 33 MHz) showing how changes in US‐nerve images may guide treatment choice.

**Methods:**

Three patients diagnosed with multifocal CIDP and followed for several years in our department were studied. Clinical evaluations, electrodiagnostic studies (EDX), laboratory analyses, and UHF‐US of the nerves were performed.

**Results:**

Nerve conduction studies (NCS) revealed severe demyelinating neuropathy with conduction blocks, reduced motor conduction velocities (MCV) in all cases, and secondary axonal degeneration in two cases. Ultrasound showed NE in the roots and nerves, with altered echogenicity and modification of internal nerve structure. A second‐line treatment was successfully started in three patients.

**Discussion:**

UHF‐US allows for the evaluation of structural changes in the nerve. NE and alterations in internal structure can be considered ultrasound markers of disease severity, guiding therapeutic decisions.

## Graphical abstract

This study highlights the potential of ultra‐high frequency ultrasound (28–33 MHz) to detect internal structural changes in enlarged nerves in multifocal CIDP. In three patients, altered echogenicity and fascicular architecture were associated with severe electrophysiological findings and guided treatment escalation. These findings support the role of UHF‐US as a tool to monitor disease severity and tailor therapy.

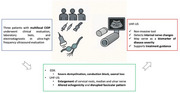

**Abbreviations**
AVM: arteriovenous malformation
CASPR1: contactin associated protein 1
CIDP: chronic inflammatory demyelinating polyneuropathy
CMT: Charcot Marie Tooth
CNTN1: contactin 1
CSA: cross‐sectional area
CSF: cerebrospinal fluid
EDX: electrodiagnostic
GM1: ganglioside 1
HbA1c: glycated hemoglobin
HIV: human immunodeficiency virus
INCAT: Inflammatory Neuropathy Cause and Treatment Group Disability Scale
IVIG: intravenous immunoglobulin
MCV: motor conduction velocity
MN: median nerve
MRC‐ss: Modified Research Council‐sum score
MRI: magnetic resonance imaging
NE: nerve enlargement
ONLS: Overall Disability Limitations Scale
PET: positron emission tomography
PLEX: plasma exchange
RTX: Rituximab
UHF‐US: ultra‐high‐frequency ultrasound
UN: ulnar nerve
US: ultrasound

## 1 Introduction

Morphological diagnostic tools, including nerve ultrasound (US) and magnetic resonance imaging (MRI) of the peripheral nerves, the plexus, and spinal roots, are emerging instruments for assessing peripheral neuropathies (Goedee et al. 2018). Nerve US may provide greater predictive value compared to other ancillary tests in diagnosing inflammatory polyneuropathies (Van den Bergh et al. 2021). There is a correlation between the normal or pathological US appearances of nerves and histological counterpart (De Silva et al. 1994; Puma et al. 2021; Ricci et al. 2022; Snoj et al. 2022). However, nerve enlargement (NE) as assessed by an increase of cross‐sectional area (CSA) is currently the only US marker of acquired and inherited demyelinating neuropathies recognized by the scientific community (Van den Bergh et al. 2021). Standardized normal values have been established for different sites of the median, ulnar, and radial nerves, as well as for the brachial plexus (Goedee et al. 2017). It is commonly accepted that acquired neuropathies typically show moderate and multifocal NE, most evident in spinal nerve roots and proximal segments of arm nerves, whereas demyelinating hereditary neuropathies show homogeneous and generalized NE throughout the nerve (Padua et al. 2018; Puma et al. 2024; Zaidman et al. 2013).

Not all studies in the literature agree that there is a direct correlation between changes in clinical severity scores and an increase in nerve size (Merola et al. 2016). Morphological changes evaluated by ultrasound (US) in chronic inflammatory demyelinating polyneuropathy (CIDP) as previously described by Padua et al. (2014) can help distinguish reversible from irreversible damage and assist in disease staging.Nerve enlargement (NE) is considered reversible and mainly reflects active inflammation and edema, whereas secondary axonal changes, fibrosis involving the endoneurium, perineurium, or epineurium, and the presence of hyperechoic scar tissue are usually indicative of irreversible damage.

According to this line of evidence, we describe three patients with multifocal CIDP who presented relapses despite conventional immunomodulatory therapy and evaluate the potential usefulness of nerve ultra‐high‐frequency probe (UHF‐US; 33 MHz) to assess disease severity. We think that US markers (e.g., NE, alteration of internal‐nerve‐structure, and echogenicity) can be used to monitor disease and guide switch therapy in non‐responder CIDP patients (Muley and Parry 2009).

## 2 Methods

### 2.1 Patients

We selected three patients followed in the Peripheral Nerve and Muscle Department at the University Hospital of Nice, France, according to the following criteria: (1) clinical, laboratory, and neurophysiological findings consistent with a diagnosis of multifocal CIDP based on EFNS/PNS criteria (Van den Bergh et al. 2021); (2) clinical follow‐up of at least 12 months; (3) experience of abrupt clinical deterioration requiring clinical assessment and re‐evaluation of therapy; and (4) nerve ultrasonography performed at the time of clinical deterioration.

All patients underwent a comprehensive diagnostic workup (Table 1) for polyneuropathy, which included blood tests, electrodiagnostic (EDX) studies, accessory salivary gland biopsy, cerebrospinal fluid analysis (CSF), and clinical evaluation. Blood tests included a complete blood count; assessments of renal and hepatic function; inflammation markers; fasting blood glucose and glycated hemoglobin (HbA1c) levels; serum vitamin B12 and methylmalonic acid; thyroid‐stimulating hormone; immunofixation of serum proteins; quantitative immunoglobulins; antinuclear antibodies; and serology for human immunodeficiency virus (HIV), hepatitis C, hepatitis B, and Borrelia burgdorferi, along with antiganglioside antibodies. Moreover, two patients (patients 1 and 3) underwent next‐generation sequencing for Charcot Marie Tooth (CMT).

**TABLE 1:** Demographic and clinical characteristics of the study participants are summarized in this table.

Patient	Gender	Age (years)	Concomitant diagnoses	CSF	Autoantibodies detected
One	Male	39	Hemorrhagic stroke	Cells: 0/mm^3^ Protein: 0.37 g/L	None
Two	Female	63	Peripheral arterial disease	Cells: 0/mm^3^ Protein: 1.43 g/L	Anti‐GM1 IgM antibodies
Three	Male	38	None	Cells: 3/mm^3^ Protein: 0.68 g/L	None

Abbreviation: CSF, cerebrospinal fluid.

**FIGURE 1:**
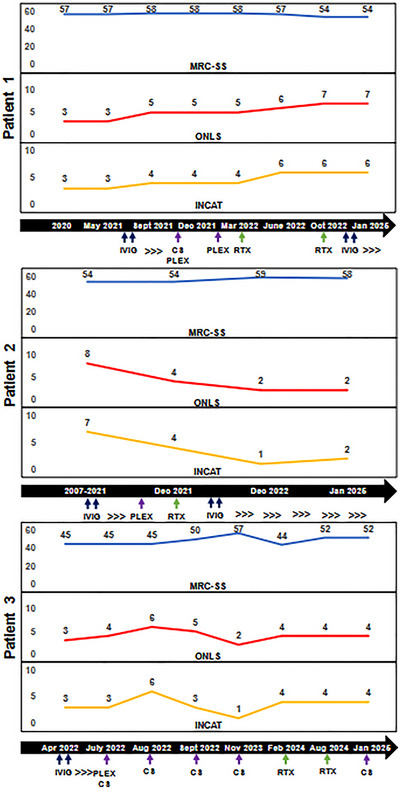
Clinical severity in the three patients, as assessed by different disability scales during follow‐up. Abbreviations: CS, corticosteroid; INCAT, Inflammatory Neuropathy Cause and Treatment; IVIG, Intravenous Immunoglobulin; MRC‐SS, Modified Research Council sum score; ONLS, Overall Neuropathy Limitations Scale; PLEX, plasma exchange; RTX, rituximab.

All patients underwent serial clinical evaluations, and neurological severity was assessed by the Modified Research Council‐sum score (MRC‐ss), the Overall Disability Limitations Scale (ONLS), and the Inflammatory Neuropathy Cause and Treatment Group Disability Scale (INCAT), performed by expert neurologists (Figure 1). Written consent was obtained from all patients, and the study was approved by local ethical committee (NCT 04978623).

### 2.2 Ultrasound Assessment

**FIGURE 2:**
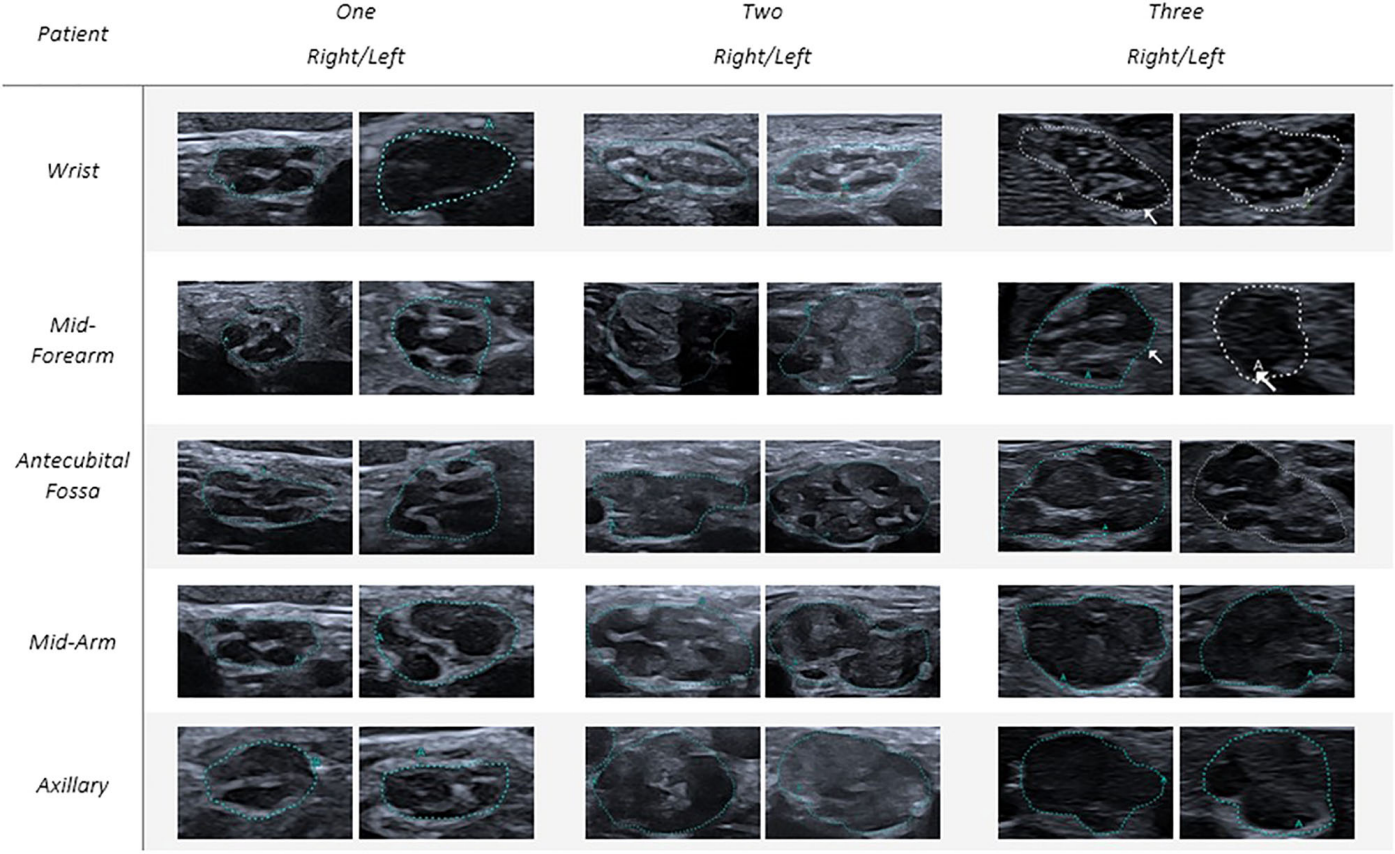
Ultrasonographic cross‐sectional area (CSA) images of the median nerve. This figure presents ultrasonographic cross‐sectional area images of the median nerve at various anatomical locations. The images illustrate the degree of nerve enlargement, highlighting any significant pathological changes. Measurements are provided to indicate the CSA at each site, facilitating a comparison of median nerve's morphology across the study subjects.

**FIGURE 3:**
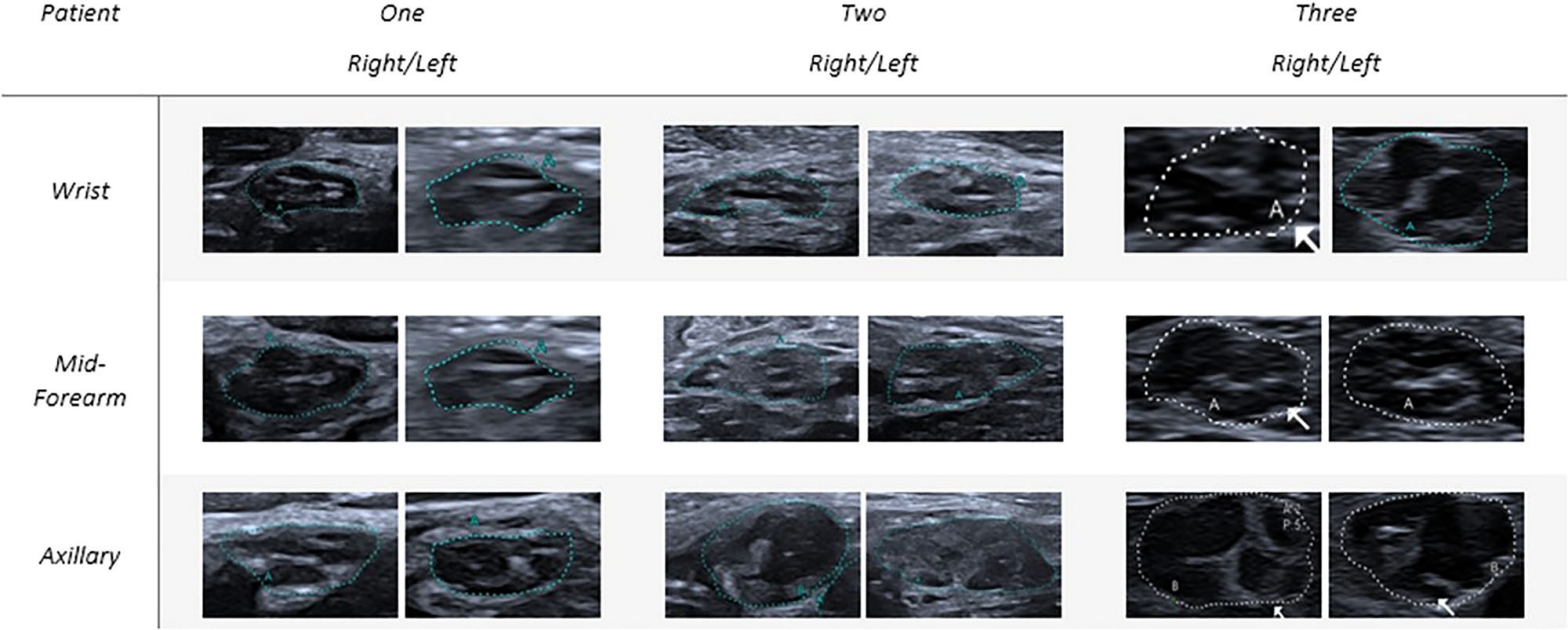
Ultrasonographic cross‐sectional area (CSA) images of the ulnar nerve. This figure presents ultrasonographic CSA images of the ulnar nerve at various anatomical locations. The images illustrate the degree of nerve enlargement, highlighting any significant pathological changes. Measurements are provided to indicate the CSA at each site, facilitating a comparison of the ulnar nerve's morphology across the study subjects.

**FIGURE 4:**
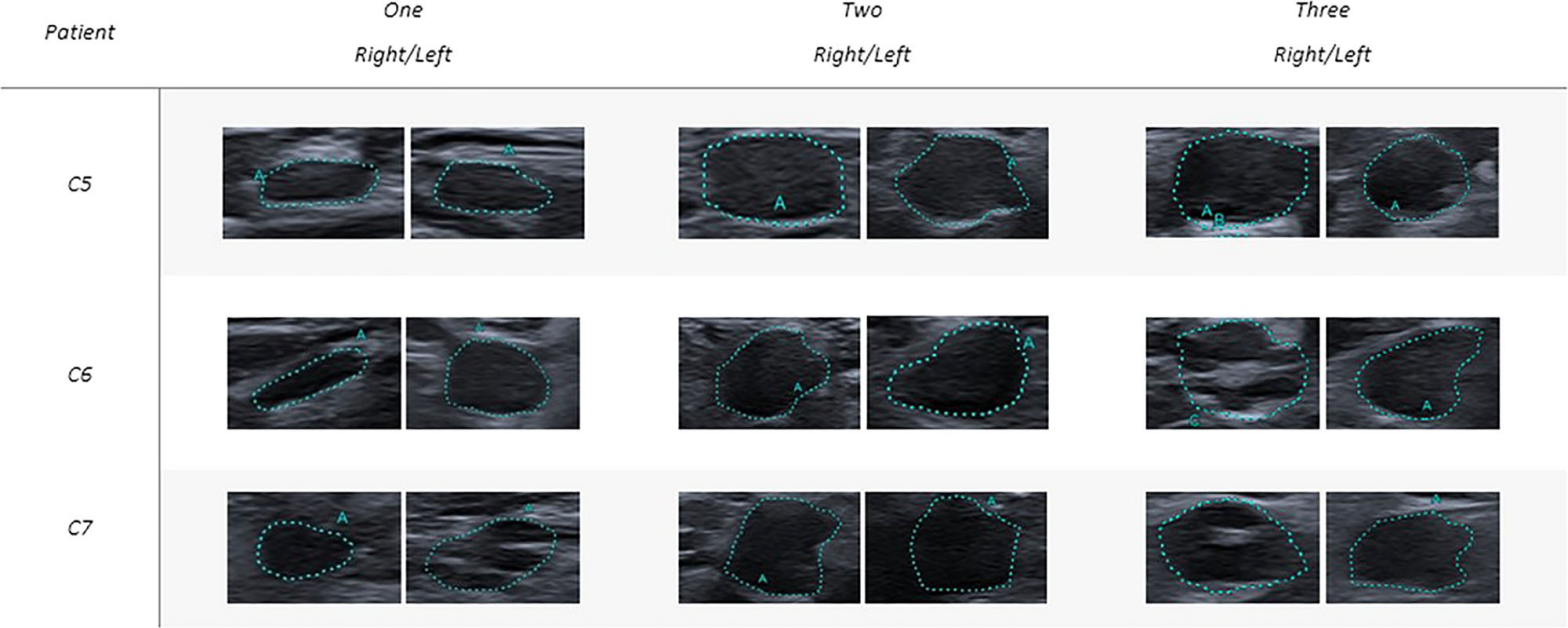
Ultrasonographic cross‐sectional area (CSA) images of the roots C5, C6, and C7. This figure presents ultrasonographic CSA images of roots C5, C6, and C7. The images illustrate the degree of nerve enlargement, highlighting any significant pathological changes. Measurements are provided to indicate the CSA at each site, facilitating a comparison of the C5, C6, and C7 roots morphology across the study subjects.

Whether clinically deteriorated, patients underwent an US evaluation of upper nerves. Nerve echography was performed by an experienced ultrasonographer using an UHF probe (i33LX9 on a Canon Aplio i800 device), adjusted perpendicular to the nerves. The assessment focused on CSA using the manual tracking method and internal structure at standardized bilateral sites: for the median nerve (MN) at the wrist, forearm (10 cm above wrist crease), antecubital fossa, mid‐arm, and axilla (Figure 2); for the ulnar nerve (UN) at the wrist, forearm (10 cm from the pisiform bone), elbow (5 cm above and below the joint), mid‐arm, and axilla (Figure 3). Additionally, the brachial plexus was evaluated at levels C5, C6, and C7 (Figure 4). First available EDX and at the time of clinical deterioration and ultrasonographic features are detailed in Tables 2 and 3, respectively.

**TABLE 2:** Findings of nerve conduction studies in right/left median and ulnar nerves.

Patient	One				Two				Three				Normal values
	First available EDX		EDX at clinical deterioration		First available EDX		EDX at clinical deterioration		First available EDX		EDX at clinical deterioration		
Median nerve	Right/left		Right/left		Right/left		Right/left		Right/left		Right/left		
MDL	55%	212%	42%	X	10%	42.5%	7.5%	32%	155%	417%	70%	162%	≤4 msec
MCV	N	39%	N	X	60%	82%	62%	58%	89%	82%	69%	67%	≥45 m/sec
cMAP Amplitude	76%	93%	N	X	N	N	N	22%	96%	86%	N	82%	≥ 5 mV
CB	No	No	No	No	Yes at elbow	No	No	Yes at Erb's Point	No	No	Yes at forearm	Yes at forearm	A^•^≥50% A^⁰^≥40%
Ulnar nerve	Right/left		Right/left		Right/left		Right/left		Right/left		Right/left		
MDL	34%	138%	14%	X	46%	54%	26%	69%	57%	420%	19%	N	≤3.5 msec
MCV	N	16%	N	X	55%	44%	58%	62%	69%	27%	58%	67%	≥45 m/sec
cMAP amplitude	42%	92%	N	X	N	N	N	18%	83%	97%	N	95%	≥ 6 mV
CB	No	Yes at axilla	Yes at Erb's point	No	No	No	Yes at Erb's point	Yes at forearm	No	No	Yes at forearm	No	A^•^≥50% A^⁰^≥40%

*Note*: Values are expressed as a percentage of normal values (*by % of normal). X indicates the absence of response, N indicates normal values.

Abbreviations: A•, amplitude; A⁰, area; CB, conduction block; cMAP, compound motor action potential; MCV, motor conduction velocity; MDL, Motor distal latency.

**TABLE 3:** Ultrasonographic features of median and ulnar nerves, C5, C6, and C7 roots in the three patients.

CSA (cm^2^)	Patient One		Patient Two		Patient Three		Normal values (cm^2^)
Sites	Right | Left		Right | Left		Right | Left		
MN wrist	0.12	**0.14**	0.12	**0.16**	**0.14**	**0.14**	0.12
MN mid‐forearm	0.08	0.08	**0.47**	**0.40**	**0.20**	**0.30**	0.11
MN antecubital fossa	**0.15**	**0.15**	**0.25**	**0.83**	**0.43**	**0.30**	0.12
MN mid‐arm	0.10	0.12	**0.39**	**0.43**	**0.29**	**0.38**	0.13
MN axillary	0.09	0.10	**0.46**	**0.49**	**0.25**	**0.22**	0.13
UN wrist	0.06	0.04	0.06	0.07	**0.10**	**0.18**	0.09
UN mid‐forearm	0.08	0.06	0.18	**0.18**	**0.13**	0.09	0.09
UN above elbow	**0.11**	0.005	**0.62**	**0.83**	**0.13**	NA	0.09
UN mid‐arm	0.08	0.06	**0.55**	**0.62**	**0.17**	NA	0.09
UN axillary	0.06	0.06	**0.49**	**0.50**	**0.26**	**0.24**	0.13
C5 root	0.06	0.06	**0.21**	**0.12**	**0.23**	**0.26**	0.11
C6 root	0.05	0.15	0.12	**0.2**	**0.25**	**0.25**	0.15
C7 root	0.05	0.14	**0.18**	**0.23**	**0.22**	**0.27**	0.17

*Note*: Bold values indicate abnormal findings.

Abbreviations: CSA, cross‐sectional area; MN, median nerve; NA, not available; NV, normal values; UN, ulnar nerve.

## 3 Results

### 3.1 Patient One

Patient one is a 39‐year‐old man with a background of hemorrhagic stroke due to a ruptured arteriovenous malformation (AVM). Over the course of 2 years, the patient progressively developed gait disturbance and a distal, asymmetric motor deficit in the upper limbs. Initial EDX indicated multifocal chronic inflammatory demyelinating polyradiculoneuropathy, characterized by motor conduction blocks in multiple segments and secondary axonal degeneration. CSF analysis showed no anomalies (Table 1). Antiganglioside antibodies, along with other blood tests, were negative.

The patient showed an initial favorable response to intravenous immunoglobulin (IVIG) for about 1 year. The patient subsequently presented a progressive clinical worsening, particularly affecting gait, motor abilities, and coordination. IVIG was replaced by plasma exchange (PLEX) without benefit. Neuropathy workup was integrated by anti‐neurofascin 155, anti‐panneurofascin, anti‐contactin 1 (CNTN1), and anti‐contactin associated protein 1 (CASPR1) antibodies and next‐generation sequencing for CMT, which were negative. A whole‐body positron emission tomography (PET) scan was also performed, with no reported anomalies. The repeated EDX revealed worsening conduction parameters (Table 2).

Nerve US during the relapse revealed discrete bilateral enlargement of the median nerve CSA at different sites (wrist, antecubital fossa) (Table 3), of right ulnar nerve above elbow and asymmetric C6 and C7 CSA (left>right). As shown in Table 3 and Figure 2, as far as the internal structure of the fascicles is concerned, a loss of normal delimitation was observed already starting from the most distal sections of the nerves. Despite secondary axonal degeneration found on EDX, US images revealed an increase in CSA along with a reduction or even disappearance of the fascicular structure. The nerve has a hypoechogenic appearance (Figure 2).

Clinical stability was achieved following the administration of rituximab (RTX), with an initial dose of 1 g administered twice, 15 days apart, and a subsequent dose of 1 g 6 months later. IVIG treatment was resumed and maintained throughout the observation period. Disability scores and subsequent therapeutic shifts are shown in Figure 1.

### 3.2 Patient Two

The second patient is a 66‐year‐old woman with a peripheral artery disease, who presented for progressive gait ataxia associated with asymmetrical distal motor weakness in all limbs.

EDX was consistent with multifocal CIDP. Blood workup was unremarkable except for positive anti‐ganglioside 1 (GM1) IgM antibodies. The CSF analysis showed an albumin‐cytological dissociation, with a protein level of 1.43 g/L (Table 1).

She was started on IVIG at 4‐week intervals, with an initial favorable clinical response. After 2 years, a rapid clinical worsening appeared with increased ataxia and loss of strength in all four limbs (Figure 1). IVIG was replaced with PLEX without clinical benefit. Diagnostic workup was supplemented through anti‐neurofascin 155, anti‐panneurofascin, anti‐CNTN1, and anti‐CASPR1 antibodies, all resulting negative. EDX (Table 2) showed more severe motor conduction slowing and diffuse conduction blocks.

Nerve US revealed an increased CSA in almost all evaluated segments of the upper limbs, measuring between two and eight times the normal values (Figures 2, 3, 4). The distribution of the enlargement appeared to be homogeneous from the forearm to the axillary region. The roots were also markedly enlarged though to a lesser extent compared to the nerve trunk. The nerves had lost their classic honeycomb structure, and fascicles were no longer easily discernible and had mixed echogenicity.

After reducing IVIG intervals unsuccessfully, the patient was started on rituximab 1 g administered in two doses 15 days apart. Following the second RTX dose during the induction regimen, the patient experienced an impressive clinical recovery. A maintenance treatment by IVIG was performed. Clinical severity and therapy during the observation period are shown in Figure 1.

### 3.3 Patient Three

A 39‐year‐old patient presented with progressive proximal muscle weakness in the left leg, which extended to the contralateral lower limb after 1 month. Over the next 5 months, the motor deficit advanced, affecting the upper limbs. Balance issues developed, necessitating bilateral support for ambulation. EDX findings were consistent with multifocal CIDP. CSF analysis revealed elevated proteins and no cellular elements (Table 1). Antibodies against gangliosides, Neurofascin 155, PanNeurofascin, CNTN1, and CASPR1 were negative, and next‐generation sequencing (NGS) for CMT disease was unremarkable. The patient underwent treatment with IVIG (three treatments of 2 g/kg) and subsequently PLEX (3 monthly sessions for 3 months) with no clinical improvement; muscle weakness and ataxia worsened, requiring wheelchair use. Diagnosis of pulmonary tuberculosis was made thereafter, requiring antitubercular therapy for 1 year. After successful treatment of the intercurrent infection, initiation of corticosteroid therapy (prednisone 60 mg/day) led to significant improvement in motor function; however, tapering the corticosteroids resulted in a clinical relapse. During the clinical relapse (see Figure 1 for severity scales), EDX showed prolonged latencies, reduced motor conduction velocities (MCVs), and numerous conduction blocks (Table 2). The US evaluation revealed a diffuse thickening of the median and ulnar nerves, with the median nerve size reaching up to four times the normal value (Figures 2 and 3). The normal honeycomb structure was lost, and fascicular anastomoses were observed, leading to the formation of a single fascicle starting from the most proximal portions of the nerve (mid‐arm, axillary) for both nerves (Table 3, Figures 2 and 3). The median nerve showed internal vascularization at the arm level. The C5, C6, and C7 nerve roots were globally thickened (Figure 4). RTX (1 g 15 days apart in first month and then after 6 months) was added to oral prednisone, ensuring a clinical stabilization. Clinical severity scales and therapeutic switch are detailed in Figure 1.

## 4 Discussion

These three clinical cases in this series presented episodes of clinical worsening following an initial period of variable stability while undergoing first‐line immunomodulatory treatment. Clinical relapse was accompanied by a worsening of EDX parameters, particularly with the appearance of conduction blocks. In all patients, we observed a reduction of cMAP (compound motor action potential) amplitude on EDX. As noted in the literature, the first US finding in inflammatory neuropathies is an increased nerve‐CSA (Goedee et al. 2019). In our cohort, patient two, and to a lesser extent patient three, exhibited a massively and homogeneously enlarged CSA, while patient one showed moderate and patchy enlargement. Nerve enlargement appears to be more significant in patients with massive demyelination on EDX evaluation (De Silva et al. 1994; Fionda et al. 2021; Puma et al. 2024), reflecting continuous demyelination–remyelination processes on the one hand and the degree of nerve inflammation on the other (Kuwabara et al. 1997; Li et al. 2019; Ricci et al. 2022).

Notably, in patient one, despite the complete absence of cMAPs in the MN and UN of the left upper limb, a moderate and uneven increase in CSA is observed in the median nerve and nerve roots, without evidence of nerve atrophy. This finding is atypical compared to what is typically seen in degenerative peripheral neuropathies, where chronic axonal loss leads to a progressive reduction in nerve volume (Stikvoort García et al. 2025). The absence of atrophy in this context supports the hypothesis of an inflammatory rather than degenerative pathology, suggesting potential for functional reversibility.

A common feature in inflammatory neuropathies is the loss of the normal internal nerve architecture, which can even be observed even in the subacute stages of the disease (Grimm et al. 2017). In a previous study, we demonstrated that the earliest US changes in CIDP include fascicle enlargement, followed by the progressive loss of the typical “honeycomb” structure (Puma et al. 2024). UHF‐US, with frequencies greater than 24 MHz, has proven particularly useful in detecting and characterizing these qualitative alterations in detail (Puma et al. 2019). These changes initially present as disorganization of the internal nerve structure, with a loss of vertical interfaces between fascicles, which tend to merge, resulting in a hypoechoic or mixed echogenic appearance that we have defined as a “magma‐like appearance.” This internal disorganization appears to be the earliest detectable alteration but, when extensive, may serve as a potential marker of disease severity. This could explain why clinical severity does not always correlate with increased CSA across all studies (Padua et al. 2014), suggesting that other factors such as fascicular structural alterations play a crucial role in determining disease severity.

The “magma‐like” appearance observed in nerve US is the result of structural changes induced by demyelination and inflammation, which lead to a loss of the normal fascicular structure and an increase in tissue heterogeneity. These changes are associated with damage that can also result in fibrosis and the formation of scar tissue.

Although this is a limited case series, we propose US markers of severity, such as increased n‐CSA, disorganization of the fascicular structure, and the hypoechoic or “magma‐like” appearance of the nerve, as useful tools to guide therapeutic decisions toward a more aggressive approach. These markers reflect acute inflammation and nerve damage and could be valuable in determining the need for second‐line immunosuppressive treatments, such as rituximab. Despite being a multifocal neuropathy, the US modifications of the nerve are bilateral and relatively symmetric, with the nerves generally and homogeneously thickened. The MN appears to be preferentially affected, aligning with previous observations reported in the literature (Ricci et al. 2022; Yoshikawa et al. 2023). Importantly, nerve roots are involved in all cases, presenting with a swollen and hypoechoic appearance. This is likely due to the fragility of the nerve–blood barrier at this level, making it more permeable to inflammatory cells (Zaidman et al. 2013).

In our series, clinical stability (patients one and three) and improvement (patient two) were achieved following treatment with rituximab, confirming its efficacy in patients with atypical CIDP (Hu et al. 2022; Menon et al. 2021). Rituximab appears to restore the effectiveness of IVIG in these patients. A marked improvement was observed in only one patient (patient two), who presented with significantly elevated CSA compared to the other two. Notably, this patient has chronic CIDP with brutal relapses, and the US revealed both chronic and acute features, reflecting the severity and complexity of the disease. Future studies should further explore the predictive value of CSA and US patterns as prognostic factors in CIDP patients. We believe that these results underscore the complementary nature of EDX and US, with the latter offering valuable insights for assessing the severity of CIDP. A limitation of this study is the limited number of patients and the lack of longitudinal ultrasonographic data for the period preceding the episode of aggravation and during the phase of clinical stability under immunosuppressive therapy.

UHF nerve US, especially those with frequencies greater than 24 MHz, appears to provide a detailed snapshot of nerve parenchyma involvement, which can aid in guiding therapeutic decisions. Timely intervention during disease exacerbation may facilitate functional recovery. In the presence of these US markers, it is advisable to promptly consider more aggressive or combined therapeutic approaches. We reiterate the hypothesis that nerve US can complement functional assessments and serve as a reliable tool in the diagnosis and monitoring of inflammatory neuropathies.

## Author Contributions

**Angela Puma**: Conceptualization, writing – original draft, writing, review and editing, visualization. **Aurora Parrotta**: writing – review and editing, visualization. **Nicolas Azulay**: writing – review and editing. **Andra Ezaru**: writing – review and editing. **Michele Cavalli**: writing – review and editing. **Mihai Ioncea**: writing – review and editing. **Luisa Villa**: writing – review and editing. **Nicolae Grecu**: writing – review and editing. **Giulia Tammam**: writing – review and editing. **Sabrina Sacconi**: writing – review and editing. **Simona Maccora**: writing – review, editing and visualization. **Charles Raffaelli**: Conceptualization, writing – original draft, writing – review and editing, visualization.

## Ethics Statement

We confirm that we have read the Journal's position on issues involved in ethical publication and affirm that this report is consistent with those guidelines.

## Conflicts of Interest

The authors declare no conflicts of interest.

## Peer Review

The peer review history for this article is available at https://publons.com/publon/10.1002/brb3.70690

## Data Availability

The data that support the findings of this study are available from the corresponding author upon reasonable request.
